# High night temperature strongly impacts TCA cycle, amino acid and polyamine biosynthetic pathways in rice in a sensitivity-dependent manner

**DOI:** 10.1093/jxb/erv352

**Published:** 2015-07-23

**Authors:** Ulrike Glaubitz, Alexander Erban, Joachim Kopka, Dirk K. Hincha, Ellen Zuther

**Affiliations:** Max-Planck-Institut für Molekulare Pflanzenphysiologie, Am Mühlenberg 1, D-14476 Potsdam, Germany

**Keywords:** Asymmetric global warming, high night temperatures, metabolite profiling, natural variation, polyamines, rice (*Oryza sativa L.*), stress tolerance, TCA cycle.

## Abstract

Global climate change combined with asymmetric warming can have detrimental effects on the yield of crop plants such as rice (*Oryza sativa L.*). Little is known about metabolic responses of rice to high night temperature (HNT) conditions. Twelve cultivars with different HNT sensitivity were used to investigate metabolic changes in the vegetative stage under HNT compared to control conditions. Central metabolism, especially TCA cycle and amino acid biosynthesis, were strongly affected particularly in sensitive cultivars. Levels of several metabolites were correlated with HNT sensitivity. Furthermore, pool sizes of some metabolites negatively correlated with HNT sensitivity under control conditions, indicating metabolic pre-adaptation in tolerant cultivars. The polyamines putrescine, spermidine and spermine showed increased abundance in sensitive cultivars under HNT conditions. Correlations between the content of polyamines and 75 other metabolites indicated metabolic shifts from correlations with sugar-phosphates and 1-kestose under control to correlations with sugars and amino and organic acids under HNT conditions. Increased expression levels of *ADC2* and *ODC1*, genes encoding enzymes catalysing the first committed steps of putrescine biosynthesis, were restricted to sensitive cultivars under HNT. Additionally, transcript levels of eight polyamine biosynthesis genes were correlated with HNT sensitivity. Responses to HNT in the vegetative stage result in distinct differences between differently responding cultivars with a dysregulation of central metabolism and an increase of polyamine biosynthesis restricted to sensitive cultivars under HNT conditions and a pre-adaptation of tolerant cultivars already under control conditions with higher levels of potentially protective compatible solutes.

## Highlight

Investigation of metabolic responses to high night temperatures in 12 differently sensitive rice cultivars showed strong effects on central metabolism in sensitive and metabolic pre-adaptation in tolerant cultivars.

## Introduction

Environmental changes have an increasing influence on crop yields all over the world due to the reduced availability of agricultural land and water resources, and especially accelerating global climate change (Lobell *et al.*, 2011). Global warming is asymmetric, with a greater increase in night compared to day temperatures, resulting in a broad decline in the diurnal temperature range (Karl *et al.*, 1991; Easterling *et al.*, 1997; Vose *et al.*, 2005). Possible causes for this trend are an increased cloud development resulting in reduced back radiation (Alward *et al.*, 1999; Braganza *et al.*, 2004; Huang *et al.*, 2006), desertification and changes in land use (Karl *et al.*, 1991; Gallo *et al.*, 1996), aerosols (Henderson-Sellers, 1992; Ramanathan *et al.*, 2007) and greater evaporation and precipitation (Chahine, 1992; Dai *et al.*, 1997).

Several studies focused on the negative effects of asymmetric warming on the yield of crop plants, such as rice (*Oryza sativa L.*) (Peng *et al.*, 2004; Cheng *et al.*, 2009; Mohammed and Tarpley, 2010; Nagarajan *et al.*, 2010; Welch *et al.*, 2010; Lobell *et al.*, 2011), identifying for example, a reduction in nitrogen and carbohydrate translocation after flowering as a possible cause of yield reduction in a high night temperature (HNT) sensitive cultivar (Shi *et al.*, 2013).

Effects of HNT during the vegetative growth phase of rice were only studied in a limited number of cultivars. They focused on growth parameters under HNT (Kanno *et al.*, 2009; Mohammed and Tarpley, 2009, 2010) or a combination of HNT with elevated CO^2^ concentrations (Cheng *et al*., 2008, 2009), on respiration and photosynthesis (Kanno *et al.*, 2009; Mohammed and Tarpley, 2009; Cheng *et al.*, 2010) or on nitrogen accumulation (Kanno *et al.*, 2009; Cheng *et al.*, 2010). Furthermore, the interactive effects of HNT and exogenously applied ascorbic acid were investigated (Shah *et al.*, 2011).

In a recent study, we classified the relative sensitivity of 12 rice cultivars from the *indica* and *japonica* subspecies using leaf chlorosis estimates (Glaubitz *et al.*, 2014). Under HNT conditions sensitive cultivars showed reduced leaf growth, while tolerant cultivars showed increased shoot fresh weight (FW) and dry weight (DW) compared to control conditions. Carbohydrate starvation as a consequence of increased respiration under HNT conditions was excluded as a reason for leaf damage and growth reduction in sensitive cultivars (Glaubitz *et al.*, 2014). Other studies reported unaffected whole plant carbon concentrations in cultivar IR72 (Cheng *et al.*, 2010), or lower sucrose and starch contents in cultivar Notohikari (Kanno *et al.*, 2009) under HNT compared to control conditions.

Despite increasing knowledge of physiological responses of rice to HNT, only a few studies provide limited insights into changes of carbohydrate and nitrogen metabolism (Cheng *et al.*, 2009; Kanno *et al.*, 2009; Glaubitz *et al.*, 2014; Xie *et al.*, 2014). Metabolomic approaches are increasingly used to elucidate the responses of crop plants to stress conditions (Nadella *et al.*, 2012; Arbona *et al.*, 2013). The parallel assessment of a broad range of metabolites can have great value as a diagnostic tool to evaluate the natural variation in metabolite composition of both wild and crop species (Fernie and Schauer, 2009) and may also provide information for future marker-assisted breeding efforts to ensure global food security.

Since the first publication on the detection of 88 metabolites in rice leaves in 2004 (Sato *et al.*, 2004), knowledge has greatly increased, recently culminating in the identification of 840 metabolites in 529 diverse rice accessions (Chen *et al.*, 2014). However, only a few reports on rice metabolomics under abiotic stress conditions were published. The investigated stresses included salinity (Zuther *et al.*, 2007; Sanchez *et al.*, 2008; Fumagalli *et al.*, 2009), drought (Fumagalli *et al.*, 2009; Degenkolbe *et al.*, 2013), submergence (Barding *et al.*, 2012), hypoxia (Narsai *et al.*, 2011) and chromium stress (Dubey *et al.*, 2010), as well as oxidative stress on rice suspension cultures (Ishikawa *et al.*, 2010), or were restricted to grain filling related metabolism (Yamakawa and Hakata, 2010).

In a previous study we classified 12 rice cultivars regarding their HNT sensitivity using chlorosis as the phenotyping parameter (Glaubitz *et al.* 2014). Here the sensitivity rank was negatively correlated with seed yield under HNT as well as with growth parameters. Whereas respiration rate was generally increased under HNT it could be excluded as a primary cause for HNT sensitivity. An analysis of monosaccharide and starch content excluded a carbon depletion under HNT but showed a higher accumulation of carbohydrates in tolerant cultivars (Glaubitz *et al.*, 2014).

Here, we used metabolite profiling by GC-MS to investigate the influence of HNT conditions on the primary metabolism of the same 12 rice cultivars with different HNT tolerance in the vegetative stage. In addition, HPLC and qRT-PCR were used for an in-depth analysis of the role of the polyamine biosynthetic pathway in HNT sensitivity of rice.

## Materials and methods

### Plant material, cultivation and HNT stress treatment

Seeds, plant material, cultivation and HNT stress treatment were the same as outlined in Glaubitz *et al.* (2014). Briefly, 12 cultivars of *Oryza sativa L.* plants from either subspecies *japonica* or *indica* were grown under control or HNT conditions in controlled climate chambers. Single seedlings were planted into pots, with 15 pots positioned together in one polypropylene box in a split-plot design, with five blocks per control or HNT treatment. Each treatment and cultivar was represented by five replicate pots that were randomized within the blocks. For control experiments the temperature was 28°C/21°C (day/night) and for HNT conditions it was 30°C/28°C (day/night). First stress symptoms (chlorosis) were visible after 7 d and fully developed after 23 d of HNT. At this time point a clear separation of all cultivars regarding their HNT sensitivity indicated by chlorosis was possible.

After 23 d of HNT treatment (48 DAS), fully expanded leaves were harvested randomized, 2−4h after the beginning of the light period and immediately frozen in liquid nitrogen. Two independent experiments were performed for both control and HNT conditions in two growth chambers.

A detailed phenotypic and physiological characterization, including measurements of photosynthetic yield, respiration, as well as carbohydrates, is described in Glaubitz *et al.* (2014). Here chlorosis was estimated as percentage of damage of all leaves using a pre-defined scale and ID numbers for every category of damage. ID numbers for score values were ranked and averaged from three independent experiments. The significance of the differences between the ranks of all cultivars was tested with a pair-wise one-way ANOVA (Glaubitz *et al.*, 2014).

### Metabolite profiling and data analysis

Metabolite profiling was performed as in Do *et al.* (2013). A fraction enriched in polar primary metabolites was prepared from 120mg ground leaf material and analysed by gas chromatography coupled to electron impact ionization-time of flight-mass spectrometry (GC/EI-TOF-MS) as described previously (Siahpoosh *et al.*, 2011).

Average mass spectral intensities over all replicate samples were calculated for every metabolite in each cultivar. Only metabolites identified in all replicate experiments (two control and two HNT experiments) were used for further analysis. Further, these averages were divided by the median of all averages of each metabolite over all cultivars and log_2_ transformed. Hierarchical clustering with Pearson correlation was applied using the analysis, visualization and data-mining software MultiExperiment Viewer (www.tm4.org/mev; version 4.5.1). PCA was performed using R (www.r-project.org)

### Polyamine analysis

Analysis of polyamines (Put, Spd and Spm) was performed according to Do *et al.* (2013) by high performance liquid chromatography (HPLC) using the dansylation method described by Smith and Davies (1985).

### Protein content

Protein content was measured using the amido black assay based on the method of Schaffner and Weissmann (1973). Ground leaf material (50mg) was extracted with 200 µl SDS buffer [50mM Tris-HCl pH 6.8, 2% (w/v) SDS, 10% (v/v) glycerol, 1% (v/v) mercaptoethanol, 12.5mM EDTA, 0.02% (w/v) bromophenol blue] and incubated at 95°C for 5min. After centrifugation the supernatant was diluted 1:2 with SDS buffer and 5 μl were transferred to a nitrocellulose membrane in three technical replicates. After air drying for 15min, the membrane was stained with amido black solution [0.1% (w/v) amido black 10B, 30% (v/v) methanol, 10% (v/v) acetic acid] for 5min on a shaker, then washed three times for 5min with 50% (v/v) methanol and air-dried for 5min. Afterwards the membrane was cut into pieces to separate stained dots, and single pieces were transferred to the wells of a 96-well plate. The membrane was then incubated in 300 µl 25 mM NaOH (in 50% (v/v) MeOH) for 5 min at room temperature, after that membrane pieces were removed. The absorbance of the solutions was measured at 620nm with a Biochrom Anthos Zenyth 340rt microplate reader (Biochrom, Cambridge, UK). Protein concentration was calculated relative to a standard curve determined with BSA.

### Quantitative RT-PCR

Quantitative RT-PCR (qRT-PCR) was performed for nine selected cultivars according to Do *et al.* (2013) with minor changes. Leaf material of three to five replicates per cultivar and treatment was homogenized using a ball mill and equal fractions were pooled to reach 100mg. Total RNA was extracted using a Trizol protocol based on the ‘single step’ method (Chomczynski and Sacchi, 2006). The quality of the cDNA was checked by qRT-PCR using primers for the 5ʹand 3ʹ ends of the *actin-1* (Os03g50890) gene (Do *et al.* 2013). Primers for genes encoding enzymes involved in polyamine biosynthesis in rice were the same as in Do *et al.* (2013).

Data were analysed using the SDS 2.0 software (Applied Biosystems) and normalized based on the expression of the housekeeping gene *actin-1*. Relative expression was calculated as 2^–ΔCt^, with ΔCt calculated by subtracting the average Ct values from three technical replicates of the housekeeping gene from the averages of the gene of interest. Fold change was calculated as log_2_ of the ratio of relative expression of genes under stress conditions to relative expression under control conditions.

### Statistics

The significance of differences between measured values was analysed using paired or unpaired, two-sided T-tests with SigmaPlot 11.0 (Systat Software GmbH, Erkrath, Germany). Significance levels are indicated as: *, 0.05>*p*>0.01; **, 0.01>*p* >0.001; ***, *p*<0.001. Correlation analysis was performed with SigmaPlot 12.3 using Spearman’s rank order correlation.

## Results

### Metabolite profiles are differentially affected by HNT conditions in different rice cultivars

To investigate the impact of high night temperature (HNT) on central metabolism, 12 previously characterized rice cultivars from either the *japonica* or *indica* subspecies (Supplementary Table S1) with different HNT tolerance were investigated under HNT (30°C day/28°C night) and control conditions (28°C day/21°C night). Classification of HNT tolerance was based on ranks of a characteristic leaf chlorosis phenotype (Glaubitz *et al.*, 2014).

Metabolite profiles were investigated for leaf blades after 23 d (48 DAS) under HNT or after 48 d under control conditions using GC-MS analysis. The vegetative stage was used to investigate symptoms of HNT sensitivity very early during development when the damage by chlorosis is already clearly separating sensitive and tolerant cultivars.

**Fig. 1.**
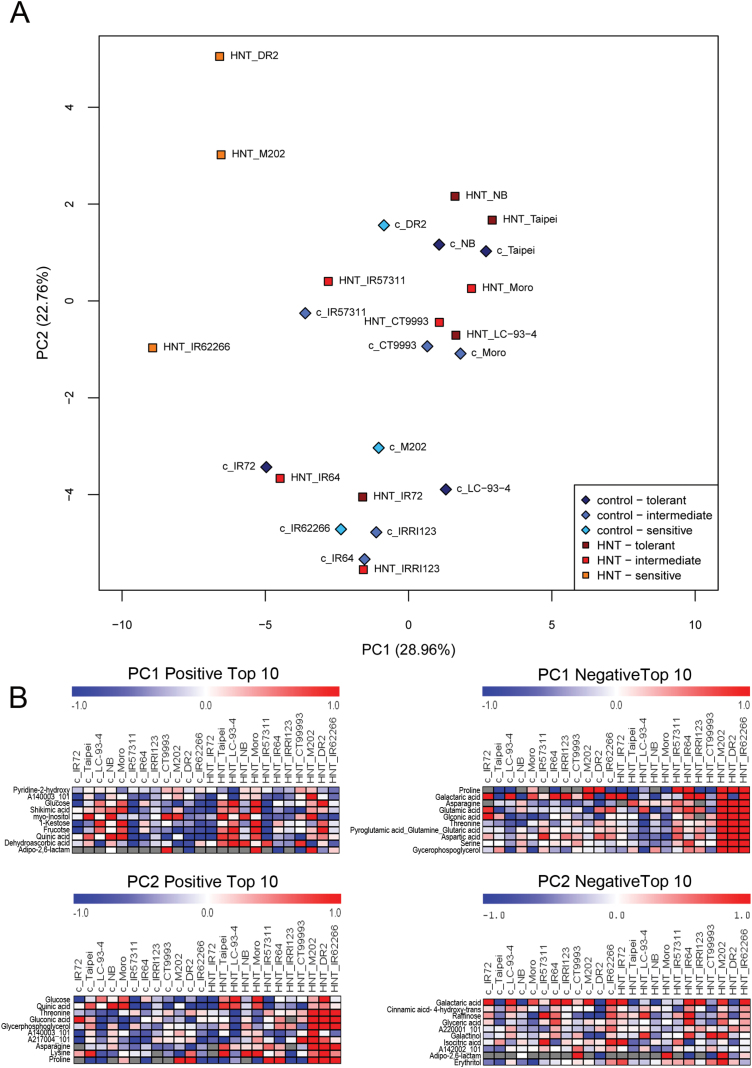
(A) Principal component analysis (PCA) of GC-MS metabolite profiles of leaves from 12 rice cultivars 48 DAS under control conditions (blue, ◊) or after 23 d (48 DAS) of HNT treatment (red, □). Tolerance groups are colour-coded (dark to light) as indicated in the figure. Scores of principal components PC1 and PC2 are shown together with the percentage of the total variance explained. (B) Metabolites with the top ten most positive and negative loadings from PC1 and PC2 are shown in panel. Metabolites with high intensities compared to the median are coloured in red, metabolites with low intensities in blue. Gray indicates a missing value.

In total, 156 metabolites were identified in two control and two HNT experiments and annotated according to the MPIMP-Golm inventory list (Kopka *et al.*, 2005) (Supplementary Table S2). Principal component analysis (PCA) and hierarchical cluster analysis (HCA) were performed on a reduced dataset of 75 metabolites common to all four experiments. Principal components (PC) 1 and 2, which together explained 51.7% of the total variance, clearly separated the metabolite profiles of the three most sensitive cultivars (M202, DR2, IR62266-42-6-2) under HNT conditions from all others (Fig. 1A). Loadings of PC1 with the highest values were adipo-2,6-lactam, dehydroascorbic acid dimer and quinic acid, while proline, galactaric acid and asparagine showed the lowest values (Fig. 1B). For PC2 proline, lysine and asparagine were the loadings with highest value and galactaric acid, 4-hydroxy-trans cinnamic acid and raffinose with the lowest (Fig. 1B). These metabolites had the highest impact on the separation of sensitive from intermediate and tolerant cultivars under HNT conditions. No distinct separation of the two subspecies could be found when both conditions were analysed together, whereas a separation of *indica* and *japonica* cultivars was apparent in a PCA using only data from control experiments (Supplementary Fig. S1). Selected representative metabolites of PC1 and PC3 with the 20 highest PC loading values responsible for this separation are shown in Supplementary Table S3.

**Fig. 2.**
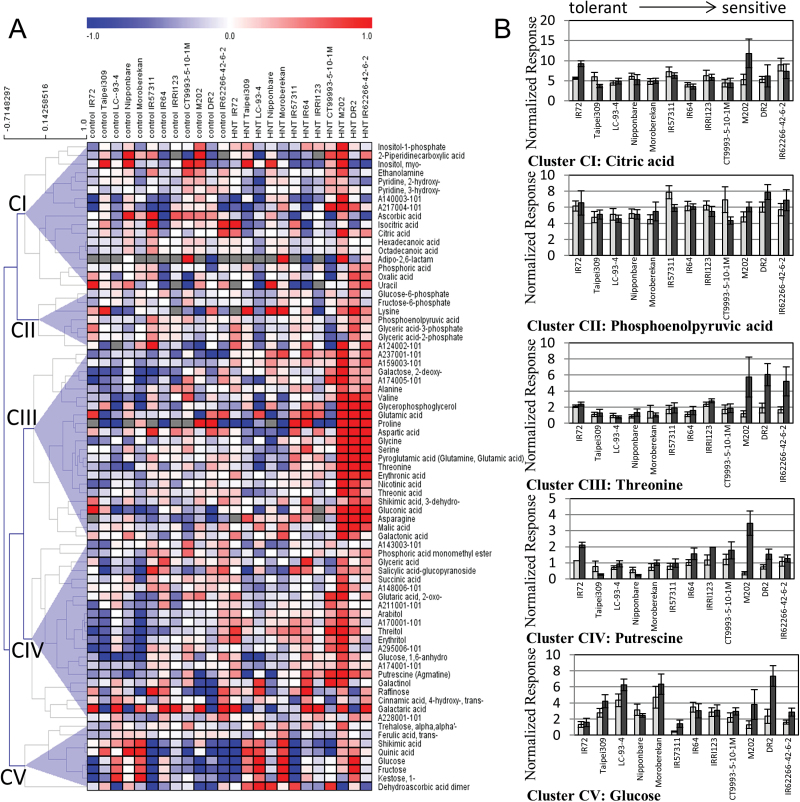
(A) Hierarchical cluster analysis (HCA) with Pearson correlation on 75 metabolites measured by GC-MS 48 DAS under control (left) and 23 d (48 DAS) of HNT (right) conditions in 12 rice cultivars indicated at the top of the figure. Metabolites with high intensities compared to the median are coloured in red, metabolites with low intensities in blue. Gray indicates a missing value. Cultivars are sorted from tolerant to sensitive (left to right). All metabolite data can be found in Supplementary Table S2. (B) Normalized responses of selected metabolites representing the clusters CI−V are shown in panel.

HCA was performed using Pearson correlation and resulted in five main clusters (CI−V) of metabolites with similar response patterns (Fig. 2A). In agreement with the results of the PCA, intermediate and sensitive cultivars showed the strongest differences in metabolite content under HNT conditions. M202 showed the highest number of metabolites with increased pool sizes upon exposure to HNT conditions. The different clusters represent different response patterns of the respective metabolites to HNT with almost no changes in CI, a minor increase of the metabolite levels in CII, a large increase in CIII in the three most sensitive cultivars and an increase in most cultivars in CIV. Pool sizes of some metabolites were only increased in sensitive cultivars, in particular those of nine amino acids (threonine is shown as an example for CIII in Fig. 2B). Also levels of the polyamine putrescine (Put), succinic and 2-oxo-glutaric acid in CIV, TCA cycle intermediates like malic acid, as well as organic acids like aspartic, pyroglutamic, erythronic, nicotinic, threonic, glucoronic and galacturonic acid (CIII) were increased in sensitive cultivars. Most of the changes in CIII and CIV occurred to a lesser extent also in intermediate cultivars, whereas the tolerant cultivars were mostly unaffected by HNT.

Fig. 2A also reveals tolerance-related differences in metabolite levels already under control conditions in CV. Leaves of most tolerant cultivars contained considerably higher levels of fructose, glucose (Fig. 2B), 1-kestose, quinic and shikimic acid compared to sensitive cultivars. Pool size changes of metabolites under HNT in comparison to control conditions also revealed the largest changes in the most sensitive cultivars (Supplementary Fig. S2, Cluster CIII-CV) with M202 again showing the highest number of positive fold changes, followed by DR2 and IR62266-42-6-2.

### Association with indicative metabolite pool sizes allows the diagnosis of relative HNT sensitivity

**Fig. 3.**
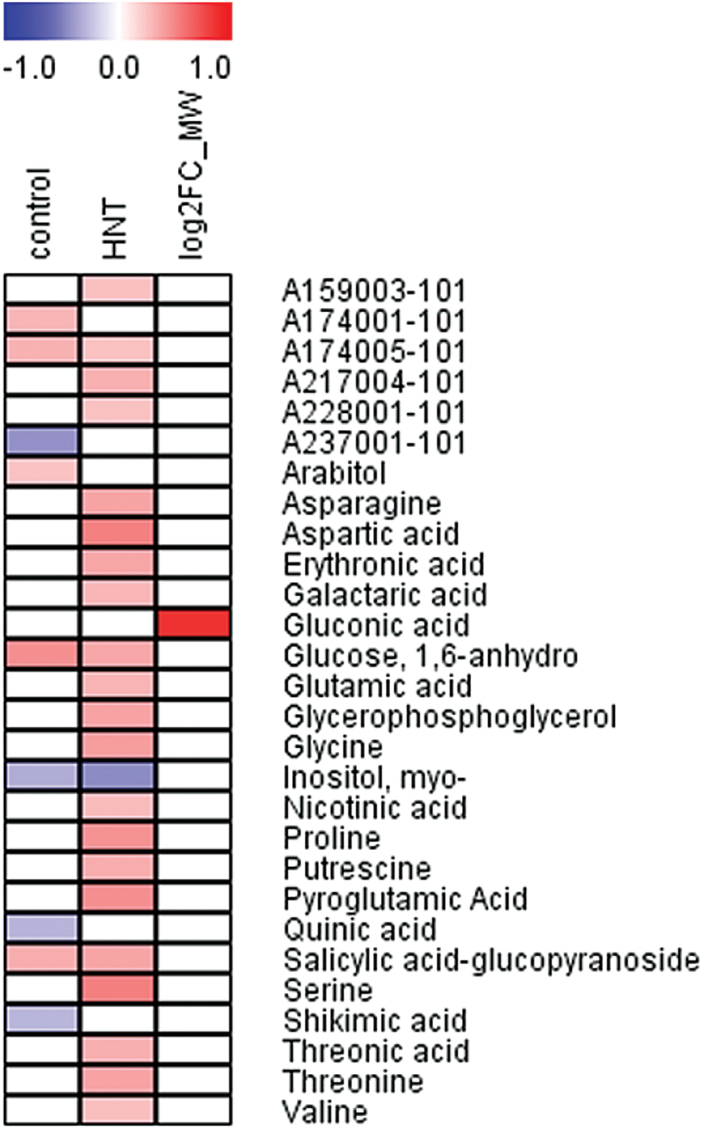
Heat map with significant Spearman correlation coefficients (  *p*<0.01) between metabolite levels and HNT sensitivity (expressed as the sensitivity rank determined from chlorosis estimates (Glaubitz *et al.*, 2014) under control and HNT conditions as well as for the log_2_ fold change in metabolite content between both conditions. Red depicts positive, blue negative correlations. Metabolites and analytes are sorted alphabetically.

To identify metabolites that may be related to HNT sensitivity, a Spearman’s rank order correlation analysis of metabolite pool sizes with HNT sensitivity was conducted (Fig. 3; Supplementary Table S4). HNT sensitivity is expressed as a rank determined by leaf chlorosis estimates, with high ranks representing HNT sensitivity (Glaubitz *et al.*, 2014). Therefore, positive correlations between metabolite levels and rank indicate high metabolite levels in sensitive and low levels in tolerant cultivars, while negative correlations reflect the opposite relationship.

Under control conditions strong positive correlations with the sensitivity rank were found for arabitol, 1,6-anhydro-glucose, salicylic acid glucopyranoside and two unknown analytes, whereas negative correlations could be shown for myo-inositol, quinic and shikimic acid and one unknown analyte. These metabolites thus represent potential predictive metabolite markers for diagnosis of HNT sensitivity that, however, still need validation under field conditions.

Under HNT conditions pool sizes of 22 of the 75 consistently detected metabolites were positively correlated with the sensitivity rank, including amino acids, organic acids and putrescine as well as four unknown analytes, which all accumulated highly in sensitive cultivars. Only myo-inositol showed a negative correlation, i.e. higher levels in tolerant cultivars. Gluconic acid was the only metabolite for which the log_2_ fold change after exposure to HNT conditions correlated significantly with the sensitivity rank, indicating high accumulation specifically in sensitive cultivars under HNT.

**Fig. 4.**
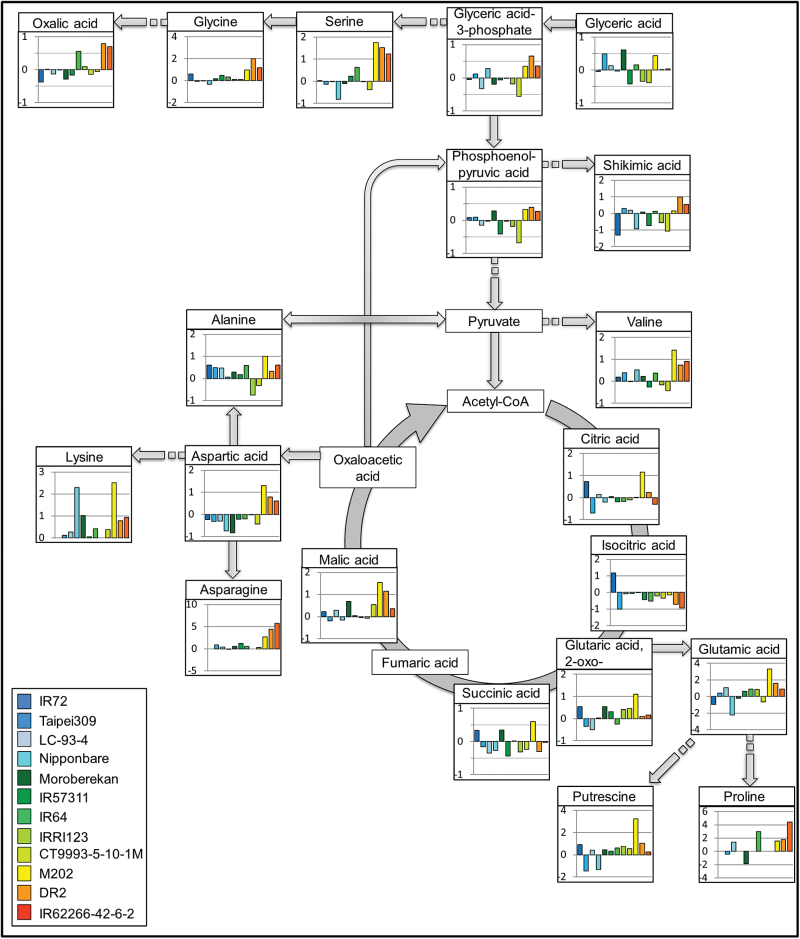
Log_2_ fold change of metabolite pool sizes of the TCA cycle and associated pathways after 23 days (48 DAS) of HNT in comparison to control conditions. Cultivars are sorted from tolerant (blue) and intermediate (green) to sensitive (yellow to red) (left to right).

When a correlation analysis was performed between metabolite pool sizes and previously collected FW data, which negatively correlated with HNT (Glaubitz *et al.*, 2014), 12 significant negative correlations (  *p*<0.01) were found under HNT conditions. An overlap of six metabolites between the two data sets including erythronic acid, glycerophosphoglycerol, pyroglutamic acid, serine, threonic acid and the unknown metabolite A217004 (not shown) pointed to an equal importance of these metabolites for a more pronounced chlorosis phenotype and reduced FW under HNT. To visualize metabolic pathways affected by HNT conditions, log_2_ fold changes in the pool sizes of metabolites of glycolysis, the TCA cycle and related pathways, mainly for the biosynthesis of amino acids, are shown in Fig. 4. Levels of nine amino acids (three from the oxaloacetate/aspartate and two from either the α-ketoglutarate, the 3-phosphoglycerate and the pyruvate family) were significantly increased in all sensitive cultivars combined with parallel increases of the TCA cycle intermediate malic acid, while in intermediate and tolerant cultivars metabolites showed smaller or no changes under HNT (Fig. 4). Glutamine could only be measured as pyroglutamic acid, a mixture of glutamine, glutamic acid and endogenous pyroglutamic acid. Although pool sizes showed a similar pattern as for glutamic acid, it was not included in the figure due to this ambiguity.

A possible reason for elevated amino acid levels after a stress treatment is increased protein degradation. Total protein content was therefore measured in the same plant material, but no significant changes were detected under HNT compared to control conditions (Supplementary Fig. S3).

### The association of polyamine metabolism with HNT sensitivity

One striking difference between sensitive and tolerant cultivars after 23 d of HNT was the increase of putrescine (Put), which was also significantly positively correlated with HNT sensitivity. Put is the first compound in the synthesis of spermidine (Spd) and spermine (Spm) and all three polyamines are known to be involved in various stress responses in plants (Alcázar *et al.*, 2010). To investigate the response of Spd and Spm to HNT conditions, polyamines were extracted independently from the previously analysed leaf material and quantified by HPLC.

**Fig. 5.**
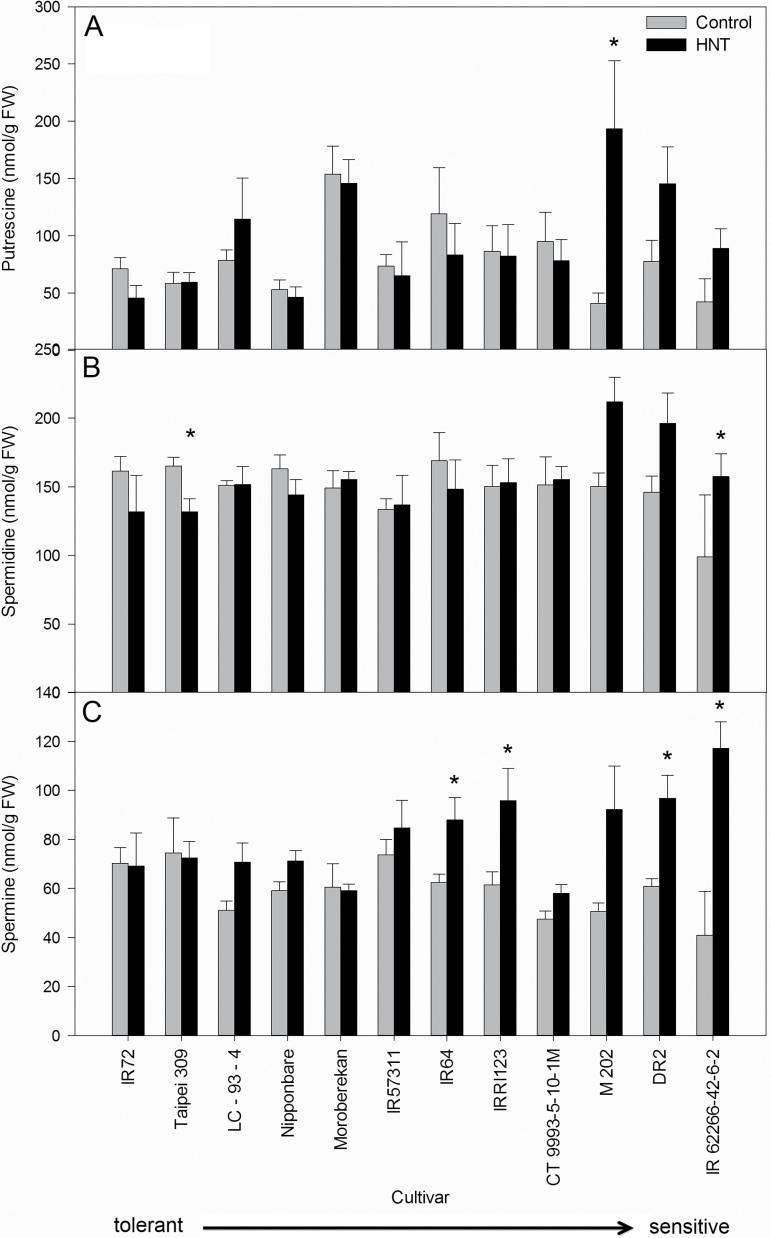
(A) Put, (B) Spd and (C) Spm content of leaves 48 DAS under control (grey columns) and 23 d (48 DAS) of HNT conditions (black columns) in 12 rice cultivars. Each value represents the average of five replicates from one experiment per condition. Cultivars are sorted from tolerant to sensitive (left to right).

Under control conditions Put levels were highly cultivar dependent with the lowest levels in the sensitive cultivar M202 (Fig. 5A). In agreement with the GC-MS analysis, Put levels were increased in sensitive cultivars under HNT conditions, with a significant increase for M202 (Fig. 5A). A similar pattern was detected for Spd with a significant increase in the sensitive cultivar IR62266-42-6-2 and a significant decrease in the tolerant cultivar Taipei 309 (Fig. 5B). Spm levels were significantly increased in the sensitive cultivars DR2 and IR62266-42-6-2 and in the intermediate cultivars IR64 and IRRI123 (Fig. 5C). Correlation analyses of polyamine levels with sensitivity rank revealed positive correlations for Put (r=0.351, *p*=0.0076), Spd (r=0.392, *p*=0.0027) and Spm (r=0.352, *p*=0.0075) under HNT but not under control conditions.

**Fig. 6.**
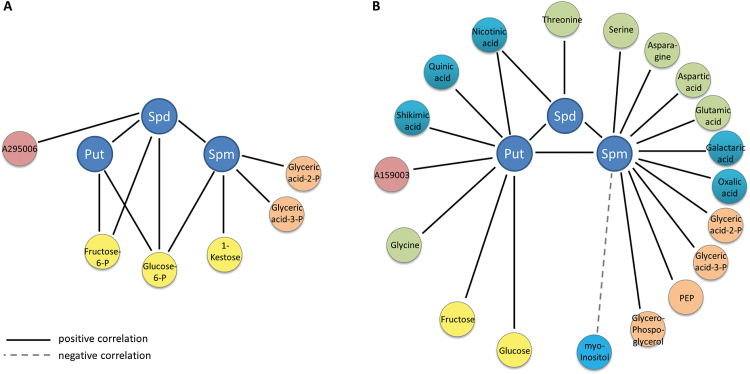
Significant (  *p*<0.05) correlations of the polyamines Put, Spd and Spm with 75 other metabolites. (A) Significant correlations under control conditions. (B) Significant correlations under HNT conditions. Solid lines indicate positive, dashed line negative correlations.

Since the content of all three polyamines was significantly correlated with the HNT sensitivity of the cultivars, we wanted to further elucidate the connection of this pathway with the central metabolism. We therefore performed a Spearman’s rank order correlation analysis of polyamine pool sizes with those of all other measured metabolites. Significant correlations differed widely between control and HNT conditions, with six and 19 correlations, respectively (Fig. 6). While Spd was positively correlated with Put and Spm under both conditions, Put was additionally correlated with Spm under HNT conditions. In addition, only the correlations of Spm with glyceric acid 2-P and glyceric acid 3-P were observed under both conditions and these metabolites were not correlated with HNT sensitivity. However, eight correlations of polyamines with amino acids or organic acids, whose levels were correlated with HNT sensitivity, were specific to HNT conditions. Further, a negative correlation between Spm and myo-inositol pool sizes was observed under HNT, where also a negative correlation of this sugar alcohol with HNT sensitivity was observed.

Interestingly, the contents of Spm and glutamic acid were correlated under HNT conditions. Glutamic acid is a precursor for polyamine biosynthesis and is derived from the TCA cycle via 2-oxo-glutaric acid (Fig. 4). In addition, serine, asparagine, aspartic acid, glyceric acid-3-P, shikimic acid and phosphoenolpyruvate (PEP) levels were correlated with Spm and glycine levels with Put levels (Fig. 6). All these compounds are connected to the TCA cycle via oxaloacetic acid and they all show high content in sensitive cultivars under HNT conditions (Fig. 4). This may indicate a higher flux through the TCA cycle in sensitive than in tolerant cultivars.

### Transcriptional regulation of polyamine biosynthesis under HNT conditions

We monitored the expression of 17 genes encoding polyamine biosynthetic enzymes to investigate whether the differences in polyamine levels in the cultivars were related to differential gene expression. Transcript abundance was measured by qRT-PCR using RNA from the same leaf samples used for polyamine analysis from eight selected cultivars (three tolerant, two intermediate and three sensitive, respectively). The selected genes encode enzymes for Put synthesis starting directly from ornithine via ornithine decarboxylase (ODC) or indirectly from arginine via agmatine including arginine decarboxylase (ADC), agmatine iminohydrolase (AIH) and N-carbamoylputrescine amidohydrolase (CPA) (Do *et al.*, 2013). Additionally, genes encoding four Spd/Spm synthases (SPD/SPM) and three S-adenosylmethionine decarboxylases (SAMDC), providing decarboxylated SAM precursors for Spd and Spm biosynthesis, were included in the analysis (Supplementary Table S5).

**Fig. 7.**
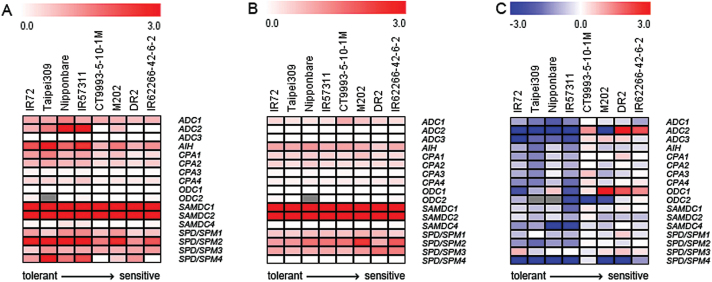
Relative expression (2^-ΔCt^) of genes encoding enzymes involved in polyamine biosynthesis in nine cultivars of rice. (A) Relative gene expression after 48 DAS under control conditions. (B) Relative gene expression after 23 days (48 DAS) of HNT conditions. (C) Log_2_ fold changes in relative gene expression after 23 days (48 DAS) of HNT in comparison to control conditions. Data represent the means of one experiment with five replicates each. Cultivars are sorted from tolerant to sensitive (left to right).

**Fig. 8.**
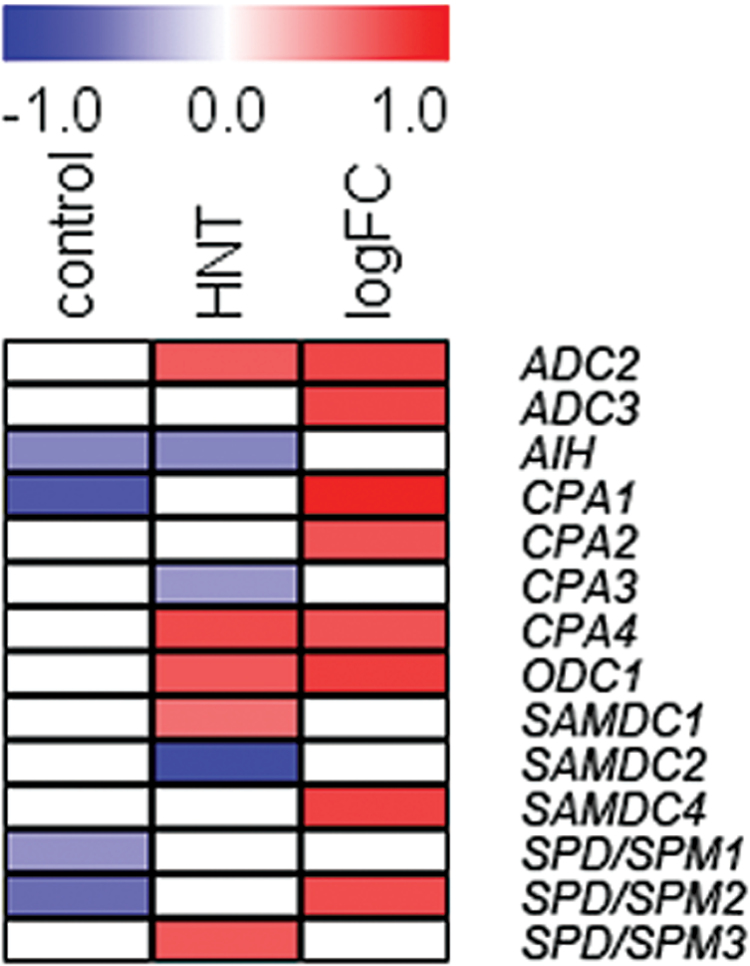
Heat map with significant Spearman correlations (p<0.05) between the expression levels of genes encoding enzymes of polyamine biosynthesis and HNT sensitivity rank (Glaubitz *et al.*, 2014) under control and HNT conditions as well as the log_2_ fold change in gene expression. Red depicts positive and blue negative correlations. Genes are sorted according to their order in the polyamine biosynthetic pathway.

ADC catalyses the first committed step of Put biosynthesis. Under control conditions, only the relative expression of *ADC2* showed a tolerance-specific pattern, with higher levels in the tolerant and two intermediate than in the sensitive cultivars (Fig. 7A; Supplementary Table S6). However, *ADC2* transcript levels were not significantly correlated with HNT sensitivity (Fig. 8, Supplementary Table S7). *AIH* showed a similar expression pattern but in this case, a significant negative correlation with HNT sensitivity was observed. Similar correlations were also found for *CPA1*, *SPD/SPM1* and *SPD/SPM2* (Figs 7A, 8). Transcript levels of all other genes showed no HNT sensitivity related patterns under control conditions.

Under HNT conditions, expression levels of all genes encoding polyamine biosynthetic enzymes decreased or were unchanged in comparison to control conditions, except for *ADC2* in the two most sensitive and *ODC1* and *SPD/SPM3* in all three sensitive cultivars (Fig. 7B, C; Supplementary Table S6). As under control conditions, the most highly expressed genes in all cultivars were *SAMDC1* and *2*, although expression of both genes was slightly reduced in most cultivars. *SAMDC1* expression was positively correlated with HNT sensitivity and the same was true for the expression levels of *ADC2*, *CPA4*, *ODC1* and *SPD/SPM3* (Fig. 8). On the other hand, negative correlations were found for the transcript abundance of *SAMDC2*, *AIH* and *CPA3* with HNT sensitivity, pointing to a higher expression in tolerant compared to sensitive cultivars.

In addition, significant positive correlations were found for eight out of the 17 investigated genes (*ADC2*, *ADC3*, *CPA1*, *CPA2*, *CPA4*, *ODC1*, *SAMDC4*, *SPD/SPM2*) between the log_2_ fold change in expression between control and HNT conditions and HNT sensitivity ranks of the cultivars (Fig. 8; Supplementary Table S7). This indicates that the strong down-regulation of polyamine biosynthetic genes in tolerant cultivars (Fig. 7C) may have functional significance for HNT tolerance in rice.

## Discussion

Composition of primary metabolites in leaves of 12 rice cultivars was determined by GC-MS analysis. Data from two experiments under HNT and control conditions each revealed 75 metabolites that could be reproducibly detected. Already under control conditions metabolic differences were observed between the cultivars that lead to a separation of the subspecies *indica* and *japonica* in a PCA analysis. GC-MS measurements on rice seeds from 100 different cultivars also showed that metabolic composition differed between *indica* and *japonica* subspecies based on 121 metabolites (Hu *et al.*, 2014). Of the 30 metabolites contributing significantly to the subspecies classification from seeds, nine were among the 10 most positive or negative loadings separating the subspecies in our analysis of leaf metabolites (asparagine, alanine, glutamic acid, putrescine, glycine, serine, gluconic acid, inositol-1-phosphate and citric acid), indicating that metabolic differences between subspecies persist independent of tissue type.

### Pool sizes of amino acids and intermediates of TCA cycle and glycolysis are correlated with HNT sensitivity

Further analysis of the leaf metabolome under both control and HNT conditions revealed differences between cultivars that were not related to subspecies but rather to HNT sensitivity. Under HNT conditions, PCA separated the three most sensitive cultivars, including *indica* and *japonica* subspecies, from all others. The most important contributors to this separation were amino and organic acids, with proline, asparagine and galactaric acid as the main responsible metabolites. Their pool sizes were also correlated with the HNT sensitivity ranks of the cultivars, together with the pool sizes of 19 other metabolites including further amino acids and intermediates of the TCA cycle and glycolysis.

Plants frequently accumulate amino acids under abiotic stress conditions (Krasensky and Jonak, 2012, and reference therein). This can be due either to an adaptive process of increased amino acid production or to stress-induced protein degradation. For instance, in rice under drought stress higher asparagine levels were predominantly found in drought sensitive cultivars (Degenkolbe *et al.*, 2013). In addition, expression of the asparagine synthetase gene correlated negatively with most performance parameters, such as total FW and DW, under both drought and control conditions. Similar correlations were also found for glutamic acid, glutamine, glycine, serine and threonine, and for the organic acids erythronic, galactonic and threonic acid, with 10- to 100-fold higher levels in sensitive compared to tolerant cultivars (Degenkolbe *et al.*, 2013). This could be related to increased protein degradation coupled with lower protein synthesis under stress (Roy-Macauley *et al.*, 1992; Munne-Bosch and Alegre, 2004; Degenkolbe *et al.*, 2009; Sanchez *et al.*, 2012). Levels of several amino acids were also highly correlated with HNT sensitivity. The strong increase in the pool sizes of amino acids in sensitive cultivars under HNT may also be related to protein degradation, as indicated by chlorosis and necrosis. However, no evidence for lower protein content in leaves of sensitive cultivars was found. In other studies, nitrogen absorption was increased under HNT in leaves and stems of rice plants (Cheng *et al.*, 2010), which could explain higher concentrations of amino acids and polyamines.

Proline content increases in response to a wide range of abiotic stresses, contributing to adaptation not only as an osmolyte, but also as a reactive oxygen species scavenger and stabilizer of DNA, membranes and proteins (recently reviewed in Krasensky and Jonak, 2012). Whereas an overexpression of Δ-1-pyrroline-5-carboxylate synthetase (P5CS), the rate-limiting enzyme in proline biosynthesis, led to increased abiotic stress tolerance in several species (for a review see Kavi Kishor and Sreenivasulu, 2014), an accumulation of proline under heat stress caused a higher heat sensitivity in *Arabidopsis* (Rizhsky *et al.*, 2004; Dobra *et al.*, 2010; Lv *et al.*, 2011). The cycling of proline synthesis and degradation necessary for balancing cellular redox potential in cytosol and plastids seems to be crucial for the antioxidant defence under stress conditions (Kavi Kishor and Sreenivasulu, 2014) and might also play a role under HNT stress conditions.

### Free polyamine levels correlated with HNT sensitivity point to a tightly regulated metabolic network with TCA cycle and amino acid synthesis

Under various stresses an accumulation of soluble nitrogen compounds, e.g. polyamines, is induced (for recent reviews, see: Groppa and Benavides, 2008; Alcázar *et al.*, 2010; Gill and Tuteja, 2010; Tiburcio *et al.*, 2014). The GC-MS analysis revealed that Put levels were increased under HNT conditions in sensitive cultivars. This was confirmed and extended to Spd and Spm by targeted HPLC analyses.

Polyamines have several functions in plants, including organogenesis, embryogenesis, floral initiation, leaf senescence, fruit development and ripening, as well as abiotic and biotic stress responses (reviewed in: Alcázar *et al.*, 2010; Tiburcio *et al.*, 2014). An involvement of polyamines in various potentially protective activities, such as protein phosphorylation (Martin-Tanguy, 2001), mediating DNA-binding activity of transcription factors (Panagiotidis *et al.*, 1995), maintenance of ion homeostasis, radical scavenging and membrane stabilization (Bouchereau *et al.*, 1999) have been described. The physiological significance of polyamine accumulation during abiotic stress responses is still under debate (Bitrián *et al.*, 2012; Gupta *et al.*, 2013) and it is unclear whether these responses are due to stress-induced injury or part of protective mechanisms (Hussain *et al.*, 2011).

Correlation analyses indicated a shift of metabolic connectivities under HNT conditions. Strikingly, correlations of polyamine levels with those of some amino and organic acids previously found to be correlated with HNT sensitivity were observed, pointing to a tightly regulated metabolic network involving the TCA cycle, amino acid and polyamine biosynthesis under HNT conditions. These analyses lead us to the hypothesis that in sensitive cultivars HNT leads to an increased metabolic flux through the TCA cycle and into biosynthetic pathways branching off from the TCA cycle at oxaloacetic acid and 2-oxo-glutaric acid. Whether this altered flux is part of an adaptive metabolic response or a sign of pathological dysregulation remains to be determined. A correlation analysis between endogeneous Put, Spd and Spm levels with metabolites from transgenic tomato fruits producing higher amounts of Spd and Spm revealed partly similar results, such as correlations with amino acid pool sizes e.g. of asparagine and threonine (Handa and Mattoo, 2010). However, most of the correlations differed from our results, indicating stress-specific responses of metabolism to HNT and not a general metabolic shift due to higher amounts of Spd and Spm.

### Tolerant cultivars are pre-adapted to HNT already under control conditions

Differences in metabolite composition that were related to HNT sensitivity were already observed under control conditions, indicating differences in metabolic composition between cultivars that may contribute to their tolerance. These included the organic acids shikimic and quinic acid, as well as the sugars glucose, fructose and 1-kestose, showing higher concentrations in tolerant than in sensitive cultivars. In addition, pool sizes of four metabolites (one unknown, myo-inositol, quinic and shikimic acid) were negatively correlated with HNT sensitivity under control conditions (i.e. higher content in tolerant cultivars), while five metabolites (two unknown, arabitol, 1,6-anhydro glucose and salicylic acid-glucopyranoside) showed positive correlations. An induction of genes encoding enzymes involved in the shikimate pathway and related aromatic amino acid metabolism is known as a response to abiotic stress (Tzin and Galili, 2010) and shikimic acid levels were also high in tolerant cultivars under HNT conditions. A further interesting point is the high content of salicylic acid-glucopyranoside in sensitive cultivars under control conditions. This compound is an inactive storage form of the plant hormone salicylic acid (SA) that is involved in the regulation of plant responses to both biotic and abiotic stresses. In addition, SA also mediates autophagy responses (Rivas-San Vicente and Plasencia, 2011). It might be hypothesized that a larger store of SA precursor in sensitive cultivars under control conditions predisposes these cultivars to an autophagy reaction under HNT conditions that would be in agreement with the chlorosis and necrosis phenotypes we have described previously (Glaubitz *et al.*, 2014). Obviously, further experiments will be needed to test this hypothesis.

Tolerant cultivars showed higher pool sizes of glucose, fructose, 1-kestose and myo-inositol, a precursor of raffinose biosynthesis. These compatible solutes play an important role in various stresses (Taji *et al.*, 2002; Valluru and Van den Ende, 2011) as both osmolytes and stabilizers of proteins and cellular structures such as membranes. Higher amounts of such protective substances may be part of a pre-adaptation strategy that prevents metabolic dysregulation under HNT conditions. Such a pre-adaptation could be simply due to the fact that more HNT-tolerant cultivars originate from warmer, more southern climates. This explanation, however, could be excluded because the origin of the most tolerant cultivar IR72 and the most sensitive cultivar IR62266-42-6-2 is the same (13°N, 121°E) according to the International Rice Genebank Collection Information System (http://www.irgcis.irri.org). An additional potentially adaptive reaction of tolerant cultivars under HNT conditions was the reduced expression of almost all genes encoding polyamine biosynthetic genes. In combination with the up-regulation of *ADC2, ODC1* and *SPD/SPM3* in sensitive cultivars, this resulted in strong correlations of expression levels with the HNT sensitivity rank. The log_2_ fold change ratios of gene expression of eight out of 17 polyamine biosynthetic genes were positively correlated with HNT sensitivity in agreement with higher polyamine levels in sensitive cultivars. Interestingly, however, the down-regulation of gene expression in tolerant cultivars was not reflected in reduced polyamine levels suggesting that maybe polyamine turn-over rates are important. The ratio of polyamine catabolism and polyamine anabolism has been suggested as the crucial factor in polyamine-mediated stress tolerance (Gupta *et al.*, 2013). It was proposed that plants may experience stress-like environments upon polyamine treatment under which the high apoplastic H_2_O_2_ generated from increased polyamine catabolism may lead to activation of developmental plant cell death (Tisi *et al.*, 2011). A further investigation of polyamine catabolism under HNT conditions and additionally enzyme activity data will be needed to test this hypothesis in the future.

In conclusion, responses to HNT in the vegetative stage in intermediate and sensitive cultivars included distinct changes in central metabolism that were not observed in tolerant cultivars, which, on the other hand, had higher content of potentially protective compatible solutes already under control conditions. This suggests that tolerant cultivars are pre-adapted to HNT conditions, thereby avoiding stress reactions and damage, while sensitive cultivars show signs of metabolic dysregulation that may be related to their visible chlorotic and necrotic phenotypes. These findings will have to be tested on field-grown plants in the future.

## Supplementary data

Supplementary data is available at *JXB* online.

Supplementary Fig. S1. Principal component analysis (PCA) of metabolite profiles measured by GC-MS after 48 DAS under control conditions in 12 cultivars of rice.

Supplementary Fig. S2. (A) Hierarchical cluster analysis (HCA) with Pearson correlation used for log_2_ fold changes of 75 metabolites measured by GC-MS after 23 d (48 DAS) of HNT in comparison to control conditions in 12 cultivars of rice. (B) Normalized responses of selected metabolites represent the response patterns of clusters CI−V.

Supplementary Fig. S3. Protein content in leaves of 12 rice cultivars after 48 DAS under control and 23 d (48 DAS) under HNT.

Supplementary Table S1. Cultivars of *Oryza sativa* L. used for HNT stress experiments.

Supplementary Table S2. Normalized metabolite profiles measured by GC-MS after 48 DAS under control and 23 d (48 DAS) under HNT conditions for all samples.

Supplementary Table S3. Top ten most positive and negative loadings of PC1 and PC3 separating *indica* and *japonica* cultivars in a PCA of metabolite profiles measured by GC-MS after 48 DAS under control conditions.

Supplementary Table S4. Spearman’s correlations for 75 metabolites with HNT sensitivity (expressed as rank of damage by chlorosis, Glaubitz *et al.* 2014) under control and HNT conditions as well as the log_2_ fold change.

Supplementary Table S5. List of genes and putative genes encoding enzymes involved in polyamine biosynthesis in rice (see Do *et al.*, 2013).

Supplementary Table S6. Relative gene expression (2^-ΔCt^) of genes encoding enzymes involved in polyamine biosynthesis after 48 DAS under control and 23 d (48 DAS) under HNT conditions and the respective log_2_ fold change in nine cultivars of rice.

Supplementary Table S7. Spearman correlation calculated for 17 genes encoding enzymes involved in polyamine biosynthesis with HNT sensitivity [expressed as rank of damage by chlorosis, (Glaubitz *et al.*, 2014)] under control and HNT conditions as well as the log_2_ fold change.

Supplementary Data
