# Supplementary material for: High night temperature strongly impacts TCA cycle, amino acid and polyamine biosynthetic pathways in rice in a sensitivity-dependent manner
Source: J Exp Bot. 2015 Jul 23;66(20):6385–97. doi: 10.1093/jxb/erv352 (PMC4588888; doi:10.1093/jxb/erv352)
Supplement: Supplementary Data [file supp_66_20_6385__index.html]

High night temperature strongly impacts TCA cycle, amino acid and polyamine biosynthetic pathways in rice in a sensitivity-dependent manner — High night temperature strongly impacts TCA cycle, amino acid and polyamine biosynthetic pathways in rice in a sensitivity-dependent manner — Supplementary Data 

# High night temperature strongly impacts TCA cycle, amino acid and polyamine biosynthetic pathways in rice in a sensitivity-dependent manner

## Supplementary Data

Data files

- Supplementary Data - Supplementary Data
- Supplementary Data - Supplementary Data
- Supplementary Data - Supplementary Data
