# Supplementary material for: High night temperature strongly impacts TCA cycle, amino acid and polyamine biosynthetic pathways in rice in a sensitivity-dependent manner
Source: J Exp Bot. 2015 Jul 23;66(20):6385–97. doi: 10.1093/jxb/erv352 (PMC4588888; doi:10.1093/jxb/erv352)
Supplement: Supplementary Data [file supp_erv352_jexbot149401_file001.pdf]

High night temperature strongly impacts TCA cycle, amino acid and polyamine biosynthetic pathways in rice in a sensitivity dependent manner

Ulrike Glaubitz, Alexander Erban, Joachim Kopka, Dirk K. Hincha, Ellen Zuther

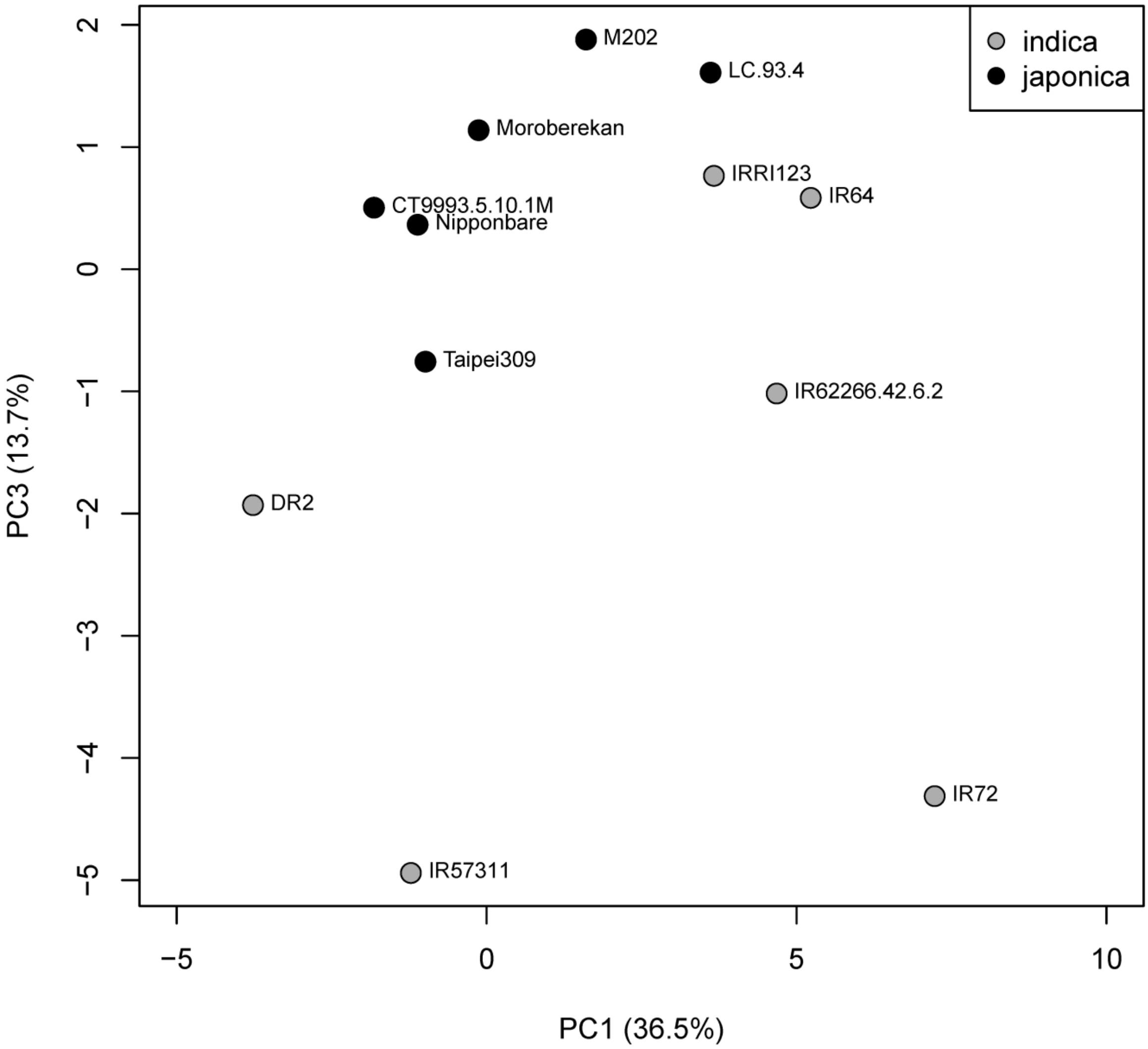

Supplementary Figure S1

Principal Component Analysis (PCA) of metabolite profiles measured by GC-MS after 48 DAS under control conditions in 12 cultivars of rice.

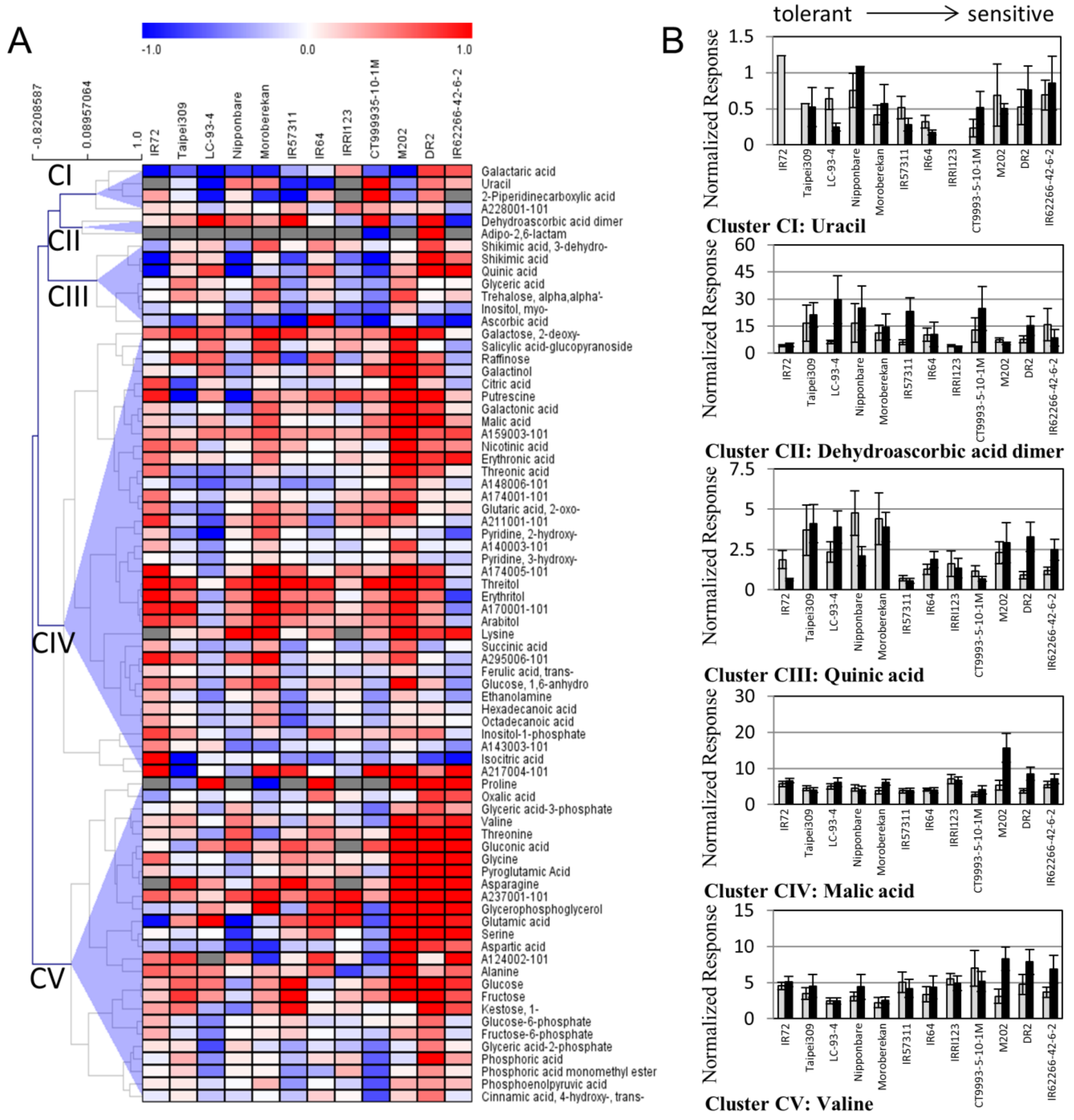

Supplementary Figure S2

(A) Hierarchical cluster analysis (HCA) with Pearson correlation used for $\log_2$ fold changes of 75 metabolites measured by GC-MS after 23 days (48 DAS) of HNT in comparison to control conditions in 12 cultivars of rice. (B) Normalized responses of selected metabolites represent the response patterns of clusters CI to CV.

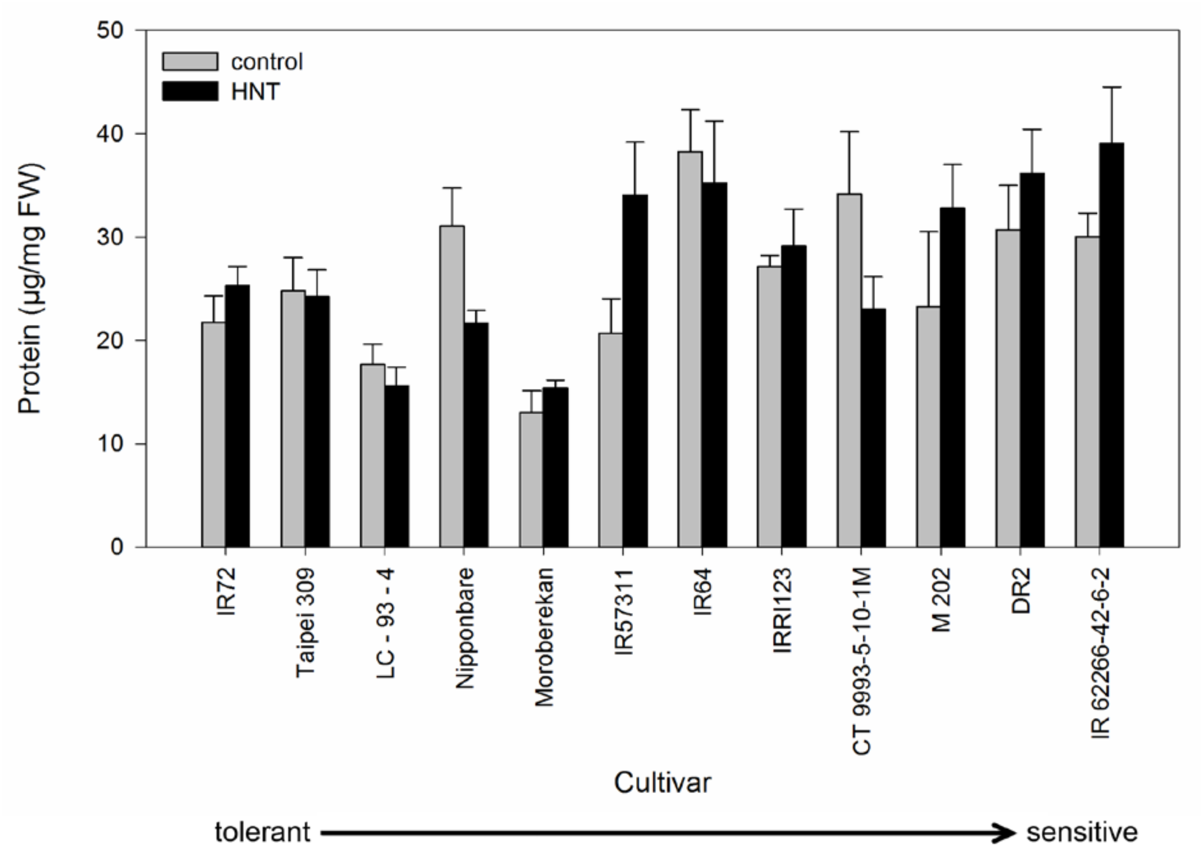

Supplementary Figure S3

Protein content in leaves of 12 rice cultivars after 48 DAS under control and 23 days (48 DAS) under HNT

Supplementary Table 1. Cultivars of *Oryza sativa* L. used for HNT stress experiments. Seeds with two origins were propagated in the latter one.

**Cultivar**	**Species**	**Origin**	**Mean Rank**
IR72	*Oryza sativa ssp. indica*	IRRI	4.1 tolerant
Taipei 309	*Oryza sativa ssp. japonica*	IRRI, MPIMP	7.5
LC-93-4	*Oryza sativa ssp. japonica*	IBT Vietnam, MPIMP	10.3
Nipponbare	*Oryza sativa ssp. japonica*	IRRI, MPIMP	10.4
Moroberekan	*Oryza sativa ssp. japonica*	IRRI	14.3
IR57311	*Oryza sativa ssp. indica*	IRRI, MPIMP	19.1
IR64	*Oryza sativa ssp. indica*	IRRI, MPIMP	20.8
IRRI123	*Oryza sativa ssp. indica*	IRRI	26.5
CT9993-5-10-1	*Oryza sativa ssp. japonica*	IRRI, MPIMP	26.5
M202	*Oryza sativa ssp. japonica*	IRRI	44.1
DR2	*Oryza sativa ssp. indica*	IBT Vietnam, MPIMP	45.2
IR62266-42-6-2	*Oryza sativa ssp. indica*	IRRI, MPIMP	45.5 sensitive

Supplementary Table 3. Top ten most positive and negative loadings of PC1 and PC3 separating *indica* and *japonica* cultivars in a PCA of metabolite profiles measured by GC-MS after 48 DAS under control conditions.

PC1	
Galactaric_acid	0.717
Adipo_26_lactam	0.218
Raffinose	0.124
Gluconic_acid	0.089
Cinnamic_acid_4_hydroxy_trans	0.086
Uracil	0.068
Glyceric_acid	0.059
Galactinol	0.049
A228001_101	0.047
Shikimic_acid_3_dehydro	0.045
Salicylic_acid_glucopyranoside	0.042
Erythritol	0.038
Glutamic_acid	0.036
Glutaric_acid_2_oxo	0.029
Asparagine	0.028
Malic_acid	0.028
Dehydroascorbic_acid_dimer	0.021
Glyceric_acid_3_phosphate	0.021
Arabitol	0.019
Glyceric_acid_2_phosphate	0.018
Inositol_1_phosphate	-0.014
Fructose_6_phosphate	-0.016
Alanine	-0.017
Octadecanoic_acid	-0.017
Aspartic_acid	-0.021
Pyridine_3_hydroxy	-0.022
Nicotinic_acid	-0.023
2_Piperidinecarboxylic_acid	-0.023
Ethanolamine	-0.025
A143003_101	-0.026
Isocitric_acid	-0.033
Threonine	-0.042
Lysine	-0.045
Valine	-0.046
Inositol_myo_	-0.047
Glycerophosphoglycerol	-0.055
Ascorbic_acid	-0.106
A140003_101	-0.134
A217004_101	-0.238
Proline	-0.529

PC3	
Adipo_26_lactam	0.380
Fructose	0.338
A217004_101	0.335
Glucose	0.319
Kestose_1_	0.294
Proline	0.197
Quinic_acid	0.188
Galactaric_acid	0.165
Shikimic_acid	0.163
A140003_101	0.152
Galactose_2_deoxy	0.088
Glycine	0.079
Inositol_myo_	0.076
A237001_101	0.072
Cinnamic_acid_4_hydroxy_trans	0.069
A174005_101	0.055
Glutaric_acid_2_oxo	0.053
Ferulic_acid_trans	0.050
A211001_101	0.049
Inositol_1_phosphate	0.045
Shikimic_acid_3_dehydro	-0.046
A124002_101	-0.050
Pyroglutamic acid;Glutamine;Glutamic acid	-0.058
Citric_acid	-0.060
Threonine	-0.061
Phosphoenolpyruvic_acid	-0.065
Glyceric_acid_2_phosphate	-0.071
Glyceric_acid_3_phosphate	-0.072
Glucose_16_anhydro	-0.073
Valine	-0.082
Ascorbic_acid	-0.083
Oxalic_acid	-0.087
Uracil	-0.095
Isocitric_acid	-0.110
Aspartic_acid	-0.114
Glutamic_acid	-0.129
Glycerophosphoglycerol	-0.131
Salicylic_acid_glucopyranoside	-0.166
Raffinose	-0.192
Lysine	-0.197

Supplementary Table 4. Spearman correlations calculated for correlations of 75 metabolites with HNT sensitivity (expressed as rank of damage by chlorosis, Glaubitz et al. 2014) under control and HNT conditions as well as the $\log_2$ fold change. P-values < 0.05 are given in italic, p-values < 0.01 are given in bold. Metabolites and analytes are sorted alphabetically.

**Metabolite**	**control**		**HNT**		**log2FC_MW**	
	correlation coefficient r	p-value	correlation coefficient r	p-value	correlation coefficient r	p-value
2-Piperidinecarboxylic acid	-0.153	0.481	0.251	0.128	0.261	0.446
A124002-101	-0.219	0.251	0.097	0.577	0.164	0.614
A140003-101	0.0791	0.42	0.174	0.0821	-0.00699	0.974
A143003-101	0.133	0.175	0.0742	0.46	-0.434	0.15
A148006-101	0.0439	0.655	0.23	*0.0206*	0.559	0.0547
A159003-101	-0.0667	0.496	0.262	**0.00821**	0.594	*0.0387*
A170001-101	0.228	*0.0187*	-0.00946	0.925	-0.399	0.189
A174001-101	0.307	**0.00144**	0.252	*0.0111*	0.217	0.484
A174005-101	0.321	**0.00093**	0.257	**0.00968**	0.014	0.956
A211001-101	0.131	0.179	0.0673	0.503	-0.0839	0.783
A217004-101	0.133	0.173	0.328	**0.000843**	0.315	0.306
A228001-101	0.154	0.115	0.257	**0.00961**	0.126	0.683
A237001-101	-0.426	**6.31E-06**	0.128	0.201	0.65	*0.0203*
A295006-101	0.311	*0.0142*	0.0225	0.863	-0.413	0.173
Adipo-2,6-lactam	-1	0.333	-0.127	0.709	1	1
Alanine	0.168	0.0849	0.178	0.0754	-0.021	0.939
Arabitol	0.254	**0.00882**	0.18	0.0711	-0.252	0.415
Ascorbic acid	-0.0164	0.884	-0.128	0.228	-0.462	0.123
Asparagine	-0.306	0.069	0.378	**0.00548**	0.612	0.0537
Aspartic acid	0.212	*0.029*	0.525	**2.29E-08**	0.65	*0.0203*
Cinnamic acid, 4-hydroxy-, trans-	-0.133	0.175	-0.0367	0.715	0.217	0.484
Citric acid	0.0271	0.782	0.0215	0.83	0.0629	0.834
Dehydroascorbic acid dimer	0.155	0.165	-0.0608	0.569	-0.322	0.295
Erythritol	0.204	*0.0365*	0.0989	0.325	-0.273	0.377
Erythronic acid	0.178	0.068	0.372	**0.00014**	0.42	0.165
Ethanolamine	0.0141	0.886	0.0534	0.595	-0.196	0.527
Ferulic acid, trans-	0.0631	0.52	-0.0496	0.622	-0.441	0.143
Fructose	-0.226	*0.0198*	-0.0171	0.865	0.413	0.173
Fructose-6-phosphate	-0.00937	0.924	0.107	0.287	0.0979	0.749
Galactaric acid	0.0958	0.371	0.305	**0.00642**	0.573	*0.0478*
Galactinol	-0.121	0.353	0.0891	0.479	0.294	0.34
Galactonic acid	-0.169	0.0853	0.0664	0.509	0.42	0.165
Galactose, 2-deoxy-	0.199	0.0567	0.119	0.233	-0.0979	0.749
Gluconic acid	-0.276	0.0638	0.205	0.137	0.864	**2E-07**
Glucose	-0.232	*0.0171*	0.00273	0.978	0.392	0.197

Metabolite	control		HNT		log2FC_MW	
	correlation coefficient r	p-value	correlation coefficient r	p-value	correlation coefficient r	p-value
Glucose, 1,6-anhydro	0.466	**6.22E-07**	0.368	**0.000166**	-0.322	0.295
Glucose-6-phosphate	-0.00945	0.923	0.141	0.159	0.196	0.527
Glutamic acid	0.11	0.264	0.31	**0.00169**	0.58	*0.0446*
Glutaric acid, 2-oxo-	0.0277	0.778	0.138	0.169	0.252	0.415
Glyceric acid	0.204	*0.0362*	0.103	0.305	-0.112	0.716
Glyceric acid-2-phosphate	-0.112	0.258	0.0741	0.47	0.014	0.956
Glyceric acid-3-phosphate	-0.0766	0.434	0.0577	0.566	0.392	0.197
Glycerophosphoglycerol	-0.0824	0.484	0.383	**0.000494**	0.594	*0.0387*
Glycine	0.0365	0.71	0.408	**2.61E-05**	0.608	*0.0333*
Hexadecanoic acid	0.0821	0.402	0.0877	0.382	-0.0769	0.8
Inositol, myo-	-0.319	**0.000906**	-0.445	**3.84E-06**	-0.196	0.527
Inositol-1-phosphate	0.0321	0.756	0.0337	0.74	-0.021	0.939
Isocitric acid	0.104	0.286	-0.158	0.115	-0.462	0.123
Kestose, 1-	-0.0961	0.327	0.026	0.796	0.224	0.47
Lysine	-0.047	0.784	0.0165	0.916	0.382	0.258
Malic acid	-0.0495	0.614	0.235	*0.018*	0.559	0.0547
Nicotinic acid	0.196	*0.0455*	0.284	**0.00406**	0.0699	0.817
Octadecanoic acid	0.106	0.28	0.112	0.264	-0.0559	0.852
Oxalic acid	-0.103	0.294	0.135	0.178	0.58	*0.0446*
Phosphoenolpyruvic acid	0.0883	0.37	0.247	*0.0137*	0.245	0.429
Phosphoric acid	-0.088	0.369	0.102	0.309	0.287	0.352
Phosphoric acid monomethyl est	0.172	0.0781	0.242	*0.0151*	0.273	0.377
Proline	0.347	0.236	0.454	**0.0056**	0.786	*0.0251*
Putrescine	0.0888	0.457	0.344	**0.00228**	0.35	0.253
Pyridine, 2-hydroxy-	0.0902	0.357	0.0481	0.633	-0.21	0.498
Pyridine, 3-hydroxy-	0.0757	0.44	0.196	*0.0498*	-0.182	0.557
Pyroglutamic Acid	0.236	*0.0155*	0.466	**1.17E-06**	0.636	*0.0242*
Quinic acid	-0.288	**0.00288**	-0.102	0.308	0.517	0.0795
Raffinose	-0.148	0.131	-0.107	0.286	-0.042	0.886
Salicylic acid-glucopyranoside	0.345	**0.000309**	0.374	**0.00013**	-0.014	0.956
Serine	-0.03	0.759	0.529	**1.59E-08**	0.58	*0.0446*
Shikimic acid	-0.281	**0.00364**	-0.166	0.0965	0.406	0.181
Shikimic acid, 3-dehydro-	0.179	0.0685	0.194	0.0526	0.301	0.329
Succinic acid	0.12	0.22	0.157	0.117	0.035	0.904
Threitol	0.185	0.0574	0.0719	0.474	-0.168	0.588
Threonic acid	0.145	0.138	0.33	**0.000778**	0.497	0.0944
Threonine	0.106	0.278	0.381	**0.000112**	0.566	0.0512
Trehalose, alpha,alpha'-	-0.0014	0.989	-0.143	0.152	-0.028	0.921
Uracil	-0.159	0.367	0.243	0.126	0.321	0.346
Valine	0.0524	0.593	0.273	**0.00585**	0.336	0.273

Supplementary Table 5. List of genes and putative genes encoding enzymes involved in polyamine biosynthesis in rice. For selection of genes see Do et al. (2013). Annotation of genes is due to the Rice Genome Annotation Project (http://rice.plantbiology.msu.edu).

**Name**	**Locus TIGR**	**Annotation TIGR**
ADC1	LOC_Os06g04070	Pyridoxal-dependent decarboxylase protein, putative, expressed
ADC2	LOC_Os04g01690	Pyridoxal-dependent decarboxylase protein, putative, expressed
ADC3	LOC_Os08g33620	Arginine decarboxylase, putative, expressed
AIH	LOC_Os04g39210	Agmatine deiminase, putative, expressed
CPA1	LOC_Os03g07910	Nitrilase, putative, expressed
CPA2	LOC_Os06g10420	Nitrilase, putative, expressed
CPA3	LOC_Os12g31830	Nitrilase, putative, expressed
CPA4	LOC_Os02g33080	N-carbamoylputrescine amidase, putative, expressed
ODC1	LOC_Os09g37120	Pyridoxal-dependent decarboxylase protein, putative, expressed
ODC2	LOC_Os04g04980	Pyridoxal-dependent decarboxylase protein, putative, expressed
SAMDC1	LOC_Os02g39795	S-adenosyl-l-methionine decarboxylase leader peptide, putative, expressed
SAMDC2	LOC_Os04g42095	S-adenosyl-l-methionine decarboxylase leader peptide, putative, expressed
SAMDC4	LOC_Os09g25625	S-adenosyl-l-methionine decarboxylase leader peptide, putative, expressed
SPD/SPM1	LOC_Os02g14190	Spermidine synthase, putative, expressed
SPD/SPM2	LOC_Os02g15550	Spermidine synthase, putative, expressed
SPD/SPM3	LOC_Os06g33710	Spermidine synthase, putative, expressed
SPD/SPM4	LOC_Os07g22600	Spermidine synthase, putative, expressed

Supplementary Table 7. Spearman correlation calculated for 17 genes encoding enzymes involved in polyamine biosynthesis with HNT sensitivity (expressed as rank of damage by chlorosis, Glaubitz et al., 2014) under control and HNT conditions as well as the log_2 fold change. P-values < 0.05 are given in italic, p-values < 0.01 are given in bold. Genes are sorted according to their order in polyamine biosynthesis.

**Gene**	**control**		**HNT**		**log2FC**	
	correlation coefficient r	p-value	correlation coefficient r	p-value	correlation coefficient r	p-value
ADC1	-0.0998	0.638	0.373	0.0717	0.429	0.26
ADC2	-0.464	0.0782	0.651	**0.00183**	0.738	*0.0287*
ADC3	-0.336	0.106	0.36	0.0829	0.738	*0.0287*
AIH	-0.47	*0.0205*	-0.468	*0.0213*	0.571	0.12
CPA1	-0.733	**0.0000002**	-0.323	0.121	0.929	**0.0000002**
CPA2	-0.368	0.076	-0.289	0.169	0.69	*0.0474*
CPA3	-0.368	0.076	-0.407	*0.048*	0.571	0.12
CPA4	-0.208	0.326	0.725	**9.33E-06**	0.69	*0.0474*
ODC1	-0.363	0.105	0.667	**0.000339**	0.786	*0.0149*
ODC2	-0.254	0.288	0.0142	0.951	0.6	0.242
SAMDC1	-0.0762	0.719	0.567	**0.00393**	0.667	0.0588
SAMDC2	-0.144	0.496	-0.835	**0.0000002**	-0.19	0.619
SAMDC4	-0.236	0.262	0.368	0.076	0.762	*0.0212*
SPD/SPM1	-0.426	*0.0381*	0.129	0.545	0.357	0.353
SPD/SPM2	-0.57	**0.00463**	0.376	0.0696	0.714	*0.0374*
SPD/SPM3	0.256	0.272	0.665	**0.000369**	0.167	0.662
SPD/SPM4	-0.488	0.0533	0.0725	0.765	0.548	0.139
